# A rat model of metabolic syndrome-related heart failure with preserved ejection fraction phenotype: pathological alterations and possible molecular mechanisms

**DOI:** 10.3389/fcvm.2023.1208370

**Published:** 2023-07-04

**Authors:** Yujiao Shi, Chunqiu Liu, Chenguang Yang, Wenbo Qiao, Yongcheng Liu, Siyu Liu, GuoJu Dong

**Affiliations:** ^1^ Department of Cardiovascular Internal Medicine, Xiyuan Hospital, Chinese Academy of Traditional Chinese Medicine, Beijing, China; ^2^ National Clinical Research Center for Chinese Medicine Cardiology, Xiyuan Hospital, Chinese Academy of Traditional Chinese Medicine, Beijing, China

**Keywords:** metabolic syndrome, heart failure with preserved ejection fraction, GDF-15, ICAM-1, VCAM-1, AKT/GSK-3β, TGF-β1/Smad

## Abstract

**Background:**

Heart failure with preserved ejection fraction (HFpEF) represents a syndrome involving multiple pathophysiologic disorders and clinical phenotypes. This complexity makes it challenging to develop a comprehensive preclinical model, which presents an obstacle to elucidating disease mechanisms and developing new drugs. Metabolic syndrome (MetS) is a major phenotype of HFpEF. Thus, we produced a rat model of the MetS-related HFpEF phenotype and explored the molecular mechanisms underpinning the observed pathological changes.

**Methods:**

A rat model of the MetS-related HFpEF phenotype was created by feeding spontaneously hypertensive rats a high-fat-salt-sugar diet and administering streptozotocin solution intraperitoneally. Subsequently, pathological changes in the rat heart and their possible molecular mechanisms were explored.

**Results:**

The HFpEF rats demonstrated primary features of MetS, such as hypertension, hyperglycemia, hyperlipidemia, insulin resistance, and cardiac anomalies, such as left ventricular (LV) remodeling and diastolic impairment, and left atrial dilation. Additionally, inflammation, myocardial hypertrophy, and fibrosis were observed in LV myocardial tissue, which may be associated with diverse cellular and molecular signaling cascades. First, the inflammatory response might be related to the overexpression of inflammatory regulators (growth differentiation factor 15 (GDF-15), intercellular adhesion molecule-1 (ICAM-1), and vascular endothelial cell adhesion molecule-1 (VCAM-1)). Secondly, phosphorylated glycogen synthase kinase 3*β* (GSK-3β) may stimulate cardiac hypertrophy, which was regulated by activated -RAC-alpha serine/threonine-protein kinase (AKT). Finally, the transforming growth factor-*β*1 (TGF-*β*1)/Smads pathway might regulate collagen production and fibroblast activation, promoting myocardial fibrosis.

**Conclusion:**

The HFpEF rat replicates the pathology and clinical presentation of human HFpEF with MetS and may be a reliable preclinical model that helps elucidate HFpEF pathogenesis and develop effective treatment strategies.

## 1. Introduction

The prevalence and mortality of heart failure (HF) with preserved ejection fraction (HFpEF), which accounts for around 50% of the HF population, are on the rise as a result of demographic aging and a growing comorbidity burden (1, 2). HFpEF represents a heterogeneous syndrome with diverse comorbidities, multiple pathophysiologic disorders, and different clinical phenotypes (3, 4). The pathological progression of HFpEF might result from a sophisticated interplay of cardiac and extra-cardiac disorders, generating marked phenotypic heterogeneity among patients (5). Consequently, this complexity and heterogeneity limit the development of comprehensive preclinical models that accurately replicate the features of human HFpEF from clinical presentation to pathophysiological changes, which presents an obstacle to elucidating disease mechanisms and poses challenges for new drug development (6). It is, therefore, imperative to create a reliable animal model of HFpEF to obtain novel insights into the underlying pathophysiological mechanisms and facilitate the development of effective therapeutic strategies.

So far, there are no well-established preclinical models of HFpEF. A certain amount of advancement has been achieved in developing animal models of HFpEF based on single risk factors related to the development and progression of HFpEF, including aging, obesity, hypertension, and diabetes (7). However, HFpEF is a systemic and complex disorder with significant phenotypic heterogeneity of pathological mechanisms and clinical features, and thus a “one-size-fits-all” model does not exist (6). Metabolic syndrome (MetS), referring to a combination of metabolic risk factors such as hypertension, hyperglycemia, hyperlipidemia, insulin resistance, and obesity, is a major comorbidity of HFpEF, with an incidence of 85% in individuals with HFpEF (8). Therefore, emerging research has begun to focus on developing animal models with MetS-related HFpEF phenotypes by mimicking metabolic disturbances. Currently, the ZSF-1 rat, a cross between Zucker diabetic fatty rats and spontaneously hypertensive heart failure rats, serves as the primary preclinical model of the MetS-associated HFpEF phenotype (9). Nevertheless, HFpEF is associated with the interaction of genetic predisposition, lifestyle factors, and a high burden of comorbidities, each contributing to various pathophysiological abnormalities. The ZSF-1 rat does not accurately mimic the human syndrome observed in HFpEF patients. Thus, there is a clear need for an animal model that mimics human HFpEF as closely as possible in all its physiological and metabolic manifestations (2).

As a result, in this study, a rat model mimicking the clinical features of the MetS-related HFpEF phenotype was established. Subsequently, the MetS and cardiac structure and function in HFpEF rats were assessed. Ultimately, we investigated the pathological changes in the rat heart (such as inflammation, hypertrophy, and fibrosis) and their underlying pathophysiological mechanisms.

## 2. Materials and methods

### 2.1. Chemicals and reagents

STZ (Cat. no. S0130) was provided by Sigma-Aldrich (Merck Millipore; Darmstadt, Germany). Total cholesterol (TC) assay kits (Cat. no. 643365), triglycerides (TG) assay kits (Cat. no. 625307), low-density lipoprotein cholesterol (LDL-C) assay kits (Cat. no. 590644), glucose (GLU) assay kits (Cat. no. 613975), and glycated serum protein (GSP) assay kits (Cat. no. 618474) were purchased from Roche Diagnostics Co. (Shanghai). Insulin assay kits (Cat. no. 20221110), atrial natriuretic peptide (ANP) assay kits (Cat. no. 20220221), B-type brain natriuretic peptide (BNP) assay kits (Cat. no. 20220217), soluble growth stimulated expression gene 2 protein (sST2) assay kits (Cat. no. 20220210BX), and galectin-3 (Gal-3) assay kits (Cat. no. 20221118) were purchased from Huaying Biotechnology Research Institute (Beijing, China). High-sensitivity C-protein (hs-CRP) assay kits (Cat. no. 20220305BX), interleukin-1β (IL-1β) assay kits (Cat. no. 20220315BX), and IL-6 assay kits (Cat. no. 20220312BX) were purchased from Xin Bosheng Biotechnology Co. (Beijing, China).

The primary antibodies included anti-growth differentiation factor 15 (GDF-15), anti-intercellular adhesion molecule-1 (ICAM-1), anti-vascular endothelial cell adhesion molecule-1 (VCAM-1), anti-phosphoinositide 3-kinase (PI3K), anti-RAC-alpha serine/threonine-protein kinase (AKT), anti-phosphorylated AKT (P-AKT), anti-glycogen synthase kinase 3*β* (GSK3*β*, Ser9), anti-P-GSK3*β*, anti-collagen type I (Coll I), anti-collagen type III (Coll III), anti-α-smooth muscle actin (*α*-SMA), anti-transforming growth factor-*β*1 (TGF-*β*1), anti-Smad2/Smad3, anti-P-Smad2/Smad3, and anti- glyceraldehyde-3-phosphate dehydrogenase (GAPDH). The antibody characteristics, including antibody names, catalog numbers, and producers, were presented in Supplementary Table S1.

### 2.2. Animals

Twenty SHR rats (SPF level, male, four weeks old, 180–200 g) and ten age-matched Wistar Kyoto (WKY) rats were purchased from Weitong Lihua Experimental Animal Technology Co. [certificate: SCXK (Beijing) 20160006, Beijing, China]. All rats were housed at the experimental animal center (Xiyuan Hospital, Chinese Academy of Chinese Medicine, China) under standard laboratory conditions (23°C and a 12-hour dark-light cycle) with free access to food and water. Twenty SHR rats were randomly assigned to the SHR and HFpEF groups (*n* = 10 per group). Ten WKY rats served as the normal control group. The rats were allowed to acclimate to the laboratory environment for four weeks before the beginning of the experiment. All animal experiments were approved by the Animal Ethics Committee of Xiyuan Hospital, Chinese Academy of Traditional Chinese Medicine (Approval no. 2021XLC008-3).

### 2.3. Establishment of the rat model with the MetS-related HFpEF phenotype

A rat model with a MetS-related HFpEF phenotype was established, which developed hypertension, hyperlipidemia, and hyperglycemia. Ten SHR rats from the HFpEF group were fed for 16 weeks a high-fat-salt-sugar diet (including 20% sucrose, 10% lard, 5% egg yolk powder, 4% NaCl, 1% cholesterol, 0.2% propylthiouracil, and 59.8% maintenance feed, supplied by Beijing Keao Xieli Feed Co.), followed by the current diet and intraperitoneal injection of STZ solution [25 mg/kg body weight (BW) per week] for eight weeks. The HFpEF group continued on their present diet for six weeks, while the WKY and SHR groups were provided with a normal maintenance diet until the end of the study. After the animal model was established, blood pressure was measured while the rat was awake. Echocardiography was done during inhalational anesthesia with isoflurane, and blood samples and heart tissue were collected.

### 2.4. Measurements of blood pressure

With a tail-cuff blood pressure device, blood pressure (systolic blood pressure (SBP) and diastolic blood pressure (DBP)) was measured non-invasively. At each time point, measurements were obtained three times, and the average result was analyzed.

### 2.5. Echocardiography analysis

A high-resolution micro-ultrasound system (Vevo 3100, FUJIFILM VisualSonics, Toronto, Canada) with an MX 250 transducer (21 MHz) was utilized to evaluate cardiac structure and function. Data analysis was conducted on Vevo LAB software version 5.7.0 (FujiFilm VisualSonics Inc.). M-mode echocardiographic tracing was used to determine left ventricle (LV) Mass, LV end-diastolic anterior wall (LVAWd), LV end-diastolic posterior wall thickness (LVPWd), LV end-diastolic internal diameter (LVEDD), LV end-diastolic volume (EDV), and LV ejection fraction (LVEF). In a four-chamber view, the maximum left atrial (LA) anterior-posterior diameter (LAAPD) and LA left-right diameter (LALRD) were measured. Regarding LV diastolic function parameters, pulsed-wave Doppler parameters (isovolumic relaxation time (IVRT) and peak mitral inflow velocity during early diastole (E)) and tissue Doppler imaging parameters (peak early diastolic mitral annular velocities (e′) and peak late diastolic mitral annular velocities (a′)) were assessed, and the E/e′ ratio was then calculated. All metrics were measured by an ultrasonographer who did not know which group each rat belonged to. Three consecutive cardiac cycles were analyzed, and an average value was determined for each metric.

### 2.6. Measurement of serum biochemical parameters

After echocardiography, blood samples were isolated to obtain serum and stored at −80 °C. TC and LDL-C were measured using an enzyme colorimetric method. TG was determined by a colorimetric method. GLU was measured with the hexokinase endpoint method. GSP was measured through a colorimetric fructosamine assay based on the nitro-blue tetrazolium reaction with ketamine. Insulin concentrations were tested with an enzyme-linked immunosorbent assay (ELISA). The index for insulin resistance (HOMA-IR) was calculated as fasting insulin (mIU/l) times fasting blood GLU (mmol/L) divided by 22.5. The fasting triglycerides and glucose (TyG) index was calculated as follows: TyG index = Ln [fasting TG (mg/dl)×fasting GLU (mg/dl)/2]. According to the manufacturer’s protocol, circulating hs-CRP, IL-1β, IL-6, ANP, BNP, sST2, and Gal-3 were determined using ELISA kits.

### 2.7. Histological evaluations

The heart tissues (*n* = 5 per group) were washed immediately with saline, then fixed in a 10% neutral formaldehyde solution at room temperature, dehydrated in an increasing series of ethanol baths, embedded in paraffin, and sectioned into 5-*μ*m sections using a microtome. Hematoxylin & eosin (H&E) (*n* = 3 per group) and Masson’s trichrome staining (*n* = 2 per group) were utilized for histological and fibrosis analysis, respectively. Moreover, the cardiomyocyte cross-sectional area (CSA, approximately 60 cardiomyocytes from 3 independent fields of view per section) and the left ventricular wall thickness (LVWT) at the midventricular level were measured to assess cardiac hypertrophy. To determine the degree of fibrosis, the collagen volume fraction (CVF) was measured with Image-Pro Plus 6.0 (Media Cybernetics, Inc.), with four distinct fields of view per section from the anterior, posterior, lateral, and septal walls of the LV; the perivascular fibrosis ratio (PFR) was determined by dividing the collagen areas deposited in the perivascular by the total vascular area.

### 2.8. Western blot analysis

The LV myocardial tissues (*n* = 4 per group) were promptly frozen in liquid nitrogen and stored at −80°C. Total protein samples were extracted using a protein extraction kit (Cat. no. WB0003; Tiande Yue Co., Beijing, China). Protein concentration was determined using a bicinchoninic acid protein quantification kit (Cat. no. WB0028, Tiangen Biotech, Beijing, China), and absorbance was measured at 570 nm using an ELISA reader (model no. Multi-Skan MK3, Thermo Fisher Scientific, America). Protein lysates were electrophoresed on a sodium dodecyl sulphate-polyacrylamide gel and transferred onto nitrocellulose membranes (NC, 0.45 um pore size; America). After blocking, membranes were incubated successively with primary antibodies (anti-GDF-15, anti-ICAM-1, anti-VCAM-1, anti-PI3K, anti-AKT, anti-P-AKT, anti-GSK-3β, anti-P-GSK-3β, anti-TGF-*β*1, anti-Smad2/3, anti-P-Smad2/Smad3, anti-Coll I, anti-Coll III, anti-α-SMA, and anti-GAPDH) and secondary antibodies of goat anti-mouse/rabbit IgG (H + L)-HRP. Signals from secondary antibodies were detected using a chemiluminescent reagent, and the image was captured using x-ray films. The results were quantified with TotalLab Quant software (Totallab, Newcastle-Upon-Tyne, UK). Finally, the relative expression of proteins was standardized using GAPDH.

### 2.9. Statistical analysis

Statistical analyses were conducted using SPSS software (version 22). All data are expressed as mean ± standard deviations. One-way analysis of variance (ANOVA) was applied to analyze the data, and *post hoc* multiple comparisons were performed with the least significant difference (LSD) test. *P*-values < 0.05, 0.01, and 0.001 indicated statistical, significant, and highly significant differences, respectively.

## 3. Results

### 3.1. Mets in HFpEF rat

**Figure 1:**
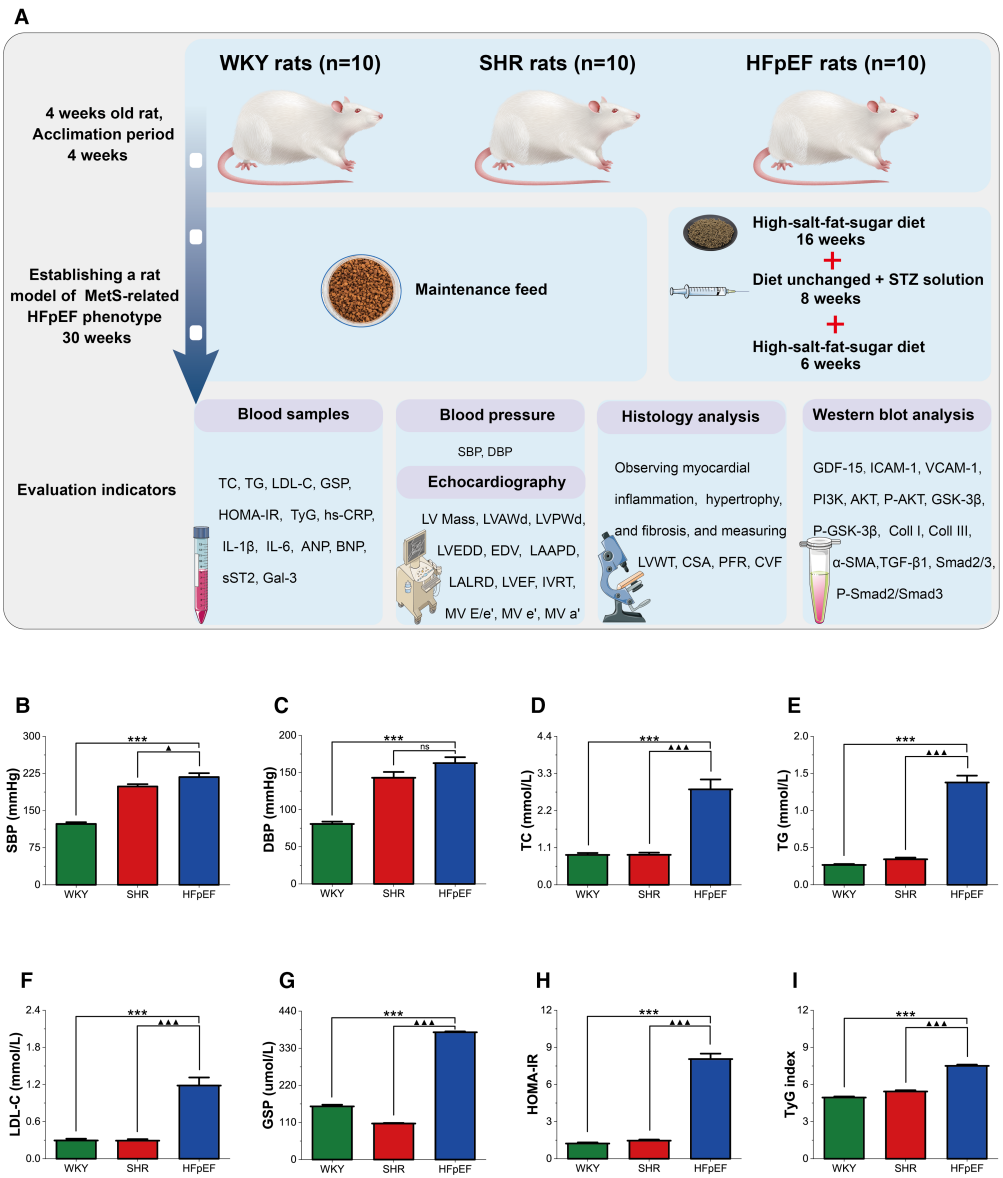
Experimental protocol and MetS in HFpEF rat. (**A**) Experimental protocol. Comparison of blood pressure (SBP and DBP) (**B,C**), plasma lipids (TC, TG, and LDL-C) (**D–F**), blood glucose (GSP) (**G**), HOMA-IR (**H**), and TyG (**I**) between the three groups. Data are presented as mean ± standard deviations, analyzed with one-way ANOVA, *P < 0.05 vs WKY;**P < 0.01 vs WKY; ***P < 0.001 vs WKY; ▴*P *< 0.05 vs. SHR, ▴▴*P *< 0.01 vs. SHR, ▴▴▴*P *< 0.001 vs SHR. WKY, Wistar Kyoto rats; SHR, spontaneously hypertensive rats; HFpEF, heart failure with preserved ejection fraction; MetS, metabolic syndrome; STZ, streptozotocin; TC, total cholesterol; TG, triglycerides; LDL-C, low-density lipoprotein cholesterol; GSP, glycated serum protein; HOMA-IR, homeostasis model assessment index for insulin resistance; TyG, fasting triglycerides and glucose index.

Figure 1A presents a schematic of the experimental procedure for the current study. The risk factors for HFpEF encompass hypertension, glucolipid dysregulation, and insulin resistance, which are also essential components of MetS. Thus, a rat model of the MetS-related HFpEF phenotype was produced by feeding SHR rats a high-fat-salt-sugar diet and administering STZ solution intraperitoneally. HFpEF rats exhibited significantly higher SBP, DBP, TC, TG, LDL-C, GSP, HOMA-IR, and TyG index than WKY rats (for all, *P *< 0.001, Figures 1B–I). When compared with SHR rats, these indicators were also significantly elevated in HFpEF rats, except for DBP (for all, *P *< 0.001). The results demonstrated that HFpEF rats (induced by risk factors of hypertension and glucolipid dysregulation) developed a cluster of clinical features of the MetS, such as hypertension, hyperglycemia, hyperlipidemia, and insulin resistance.

### 3.2. Cardiac structure and function by echocardiography in HFpEF rat

**Figure 2:**
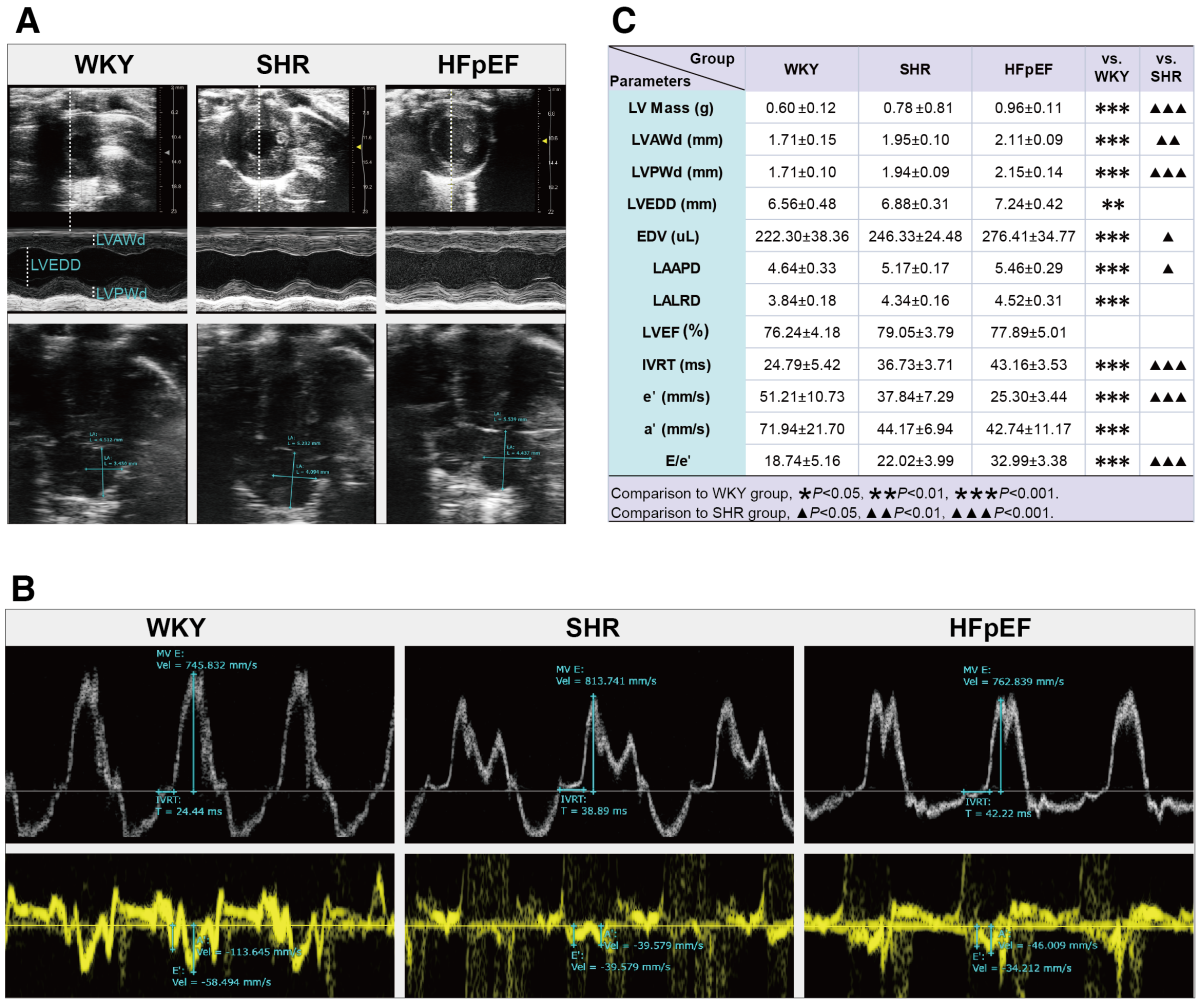
Cardiac structure and function by echocardiography in HFpEF rat. (**A**) Representative images of M-mode echocardiography (top), four-chambered heart (bottom). (**B**) pulsed-wave Doppler parameters (top) and tissue Doppler imaging (bottom). (**C**) Comparison of LV structure (LV Mass, LVAWd, LVPWd, LVEDD, and EDV), LA structure (LAAPD and LALRD), and LV systolic (LVEF) and diastolic function (IVRT, e′, a′, and E/e′) between the three groups. Data are presented as mean ± standard deviations, analyzed with one-way ANOVA, *P < 0.05 vs. WKY;**P < 0.01vs WKY; ***P < 0.001 vs WKY; ▴*P *< 0.05 vs. SHR, ▴▴*P *< 0.01 vs. SHR, ▴▴▴*P *< 0.001 vs SHR. LV, left ventricle; LVAWd, LV end-diastolic anterior wall; LVPWd, LV end-diastolic posterior wall thickness; LVEDD, LV end-diastolic internal diameter; EDV, end-diastolic volume; LA, left atrial; LAAPD, LA anterior-posterior diameter; LALRD, LA left-right diameter; LVEF, LV ejection fraction; IVRT, isovolumic relaxation time; E, peak mitral inflow velocity during early diastole; e′, peak early diastolic mitral annular velocities; a′, peak late diastolic mitral annular velocities.

Structurally. Compared to WKY rats, parameters related to cardiac structure, such as LV Mass, LVAWd, LVPWd, LVEDD, EDV, LAAPD, and LALRD, were significantly increased in HFpEF rats (for all, *P* < 0.01 or 0.001, Figures 2A,C). When compared with SHR rats, a marked increase in LV Mass, EDV, LVWd, LVPWd, and LAAPD was observed in HFpEF rats (for all, *P* < 0.05, 0.01, or 0.001). Therefore, HFpEF rats exhibited cardiac remodeling, including increased LV mass, thickened LV walls, and enlarged LA and LV internal diameters.

As for cardiac function, the HFpEF rats had a preserved LVEF, as shown by the fact that there were no statistical differences in LVEF values among the three groups. Compared with the WKY rats, parameters associated with LV diastolic function were abnormal in HFpEF rats, with markedly increased IVRT and the ratio of E/e′, as well as significantly decreased a′ and e′ (for all, *P* < 0.001, Figures 2B,C). Similar results were obtained when compared with SHR rats. The results showed that HFpEF rats developed LV diastolic dysfunction, manifested by prolonged isovolumic diastole, elevated LV filling pressures, and decreased LV relaxation and compliance.

### 3.3. Inflammation in HFpEF rat

Chronic systemic inflammation is a central pathological mechanism of HFpEF (10). GDF-15 is an inflammatory cytokine secreted in response to inflammatory stress. Cell adhesion molecules, especially ICAM-1 and VCAM-1, mediate leukocyte recruitment to the inflammation site.

**Figure 3:**
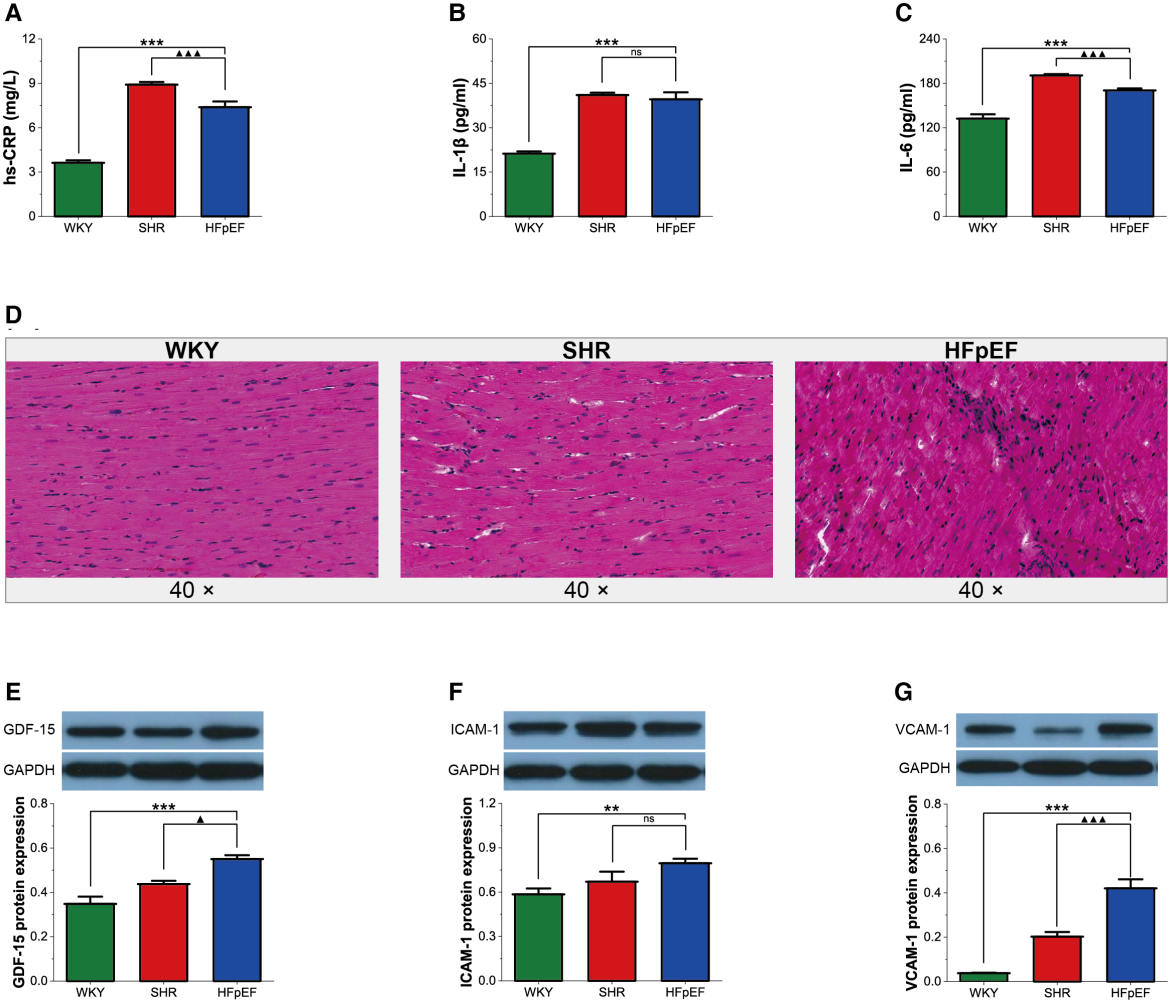
Inflammation in HFpEF rat. (**A–C**) Comparison of hs-CRP, IL-1β and IL-6 between the three groups. (**D**) Representative images of HE-stained sections of LV myocardial tissue from each group (40×): HFpEF rats developed chronic myocardial inflammation, with local inflammatory cell infiltration, predominantly lymphocytes, monocytes, and macrophages, compared to WKY and SHR rats. (**E–G**) Comparison of protein expression of GDF-15, ICAM-1, and VCAM-1 between the three groups. Data are presented as mean ± standard deviations, analyzed with one-way ANOVA, *P < 0.05 vs. WKY;**P < 0.01 vs WKY; ***P < 0.001 vs WKY; ▴*P *< 0.05 vs. SHR, ▴▴*P *< 0.01 vs. SHR, ▴▴▴*P *< 0.001 vs SHR. hs-CRP, high-sensitivity C-protein; IL-1β, interleukin-1β; IL-6, interleukin-6; GDF-15, growth differentiation factor 15; ICAM-1, intercellular adhesion molecule-1; VCAM-1, vascular endothelial cell adhesion molecule-1.

In the present research, although serum inflammatory markers (hs-CRP and IL-6) in HFpEF rats were lower than in SHR rats (for all, *P* < 0.001, Figures 3A,C), serum hs-CRP, IL-1β, and IL-6 in HFpEF rats were statistically higher than in WKY rats (for all, *P* < 0.001, Figures 3A–C). The results suggested that HFpEF rats exhibited a significant systemic inflammatory state. In addition, compared with WKY and SHR rats, a large infiltration of inflammatory cells (mainly lymphocytes, plasma cells, and macrophages) was observed in the myocardium of HFpEF rats (Figure 3D), suggesting a local inflammation response in the myocardium of HFpEF rats. Furthermore, protein expression of GDF-15, ICAM-1, and VCAM-1 was significantly higher in HFpEF rats than in WKY rats (for all, *P *< 0.01 or 0.001, Figures 3E–G), and protein expression of GDF-15 and VCAM-1 was significantly higher than in SHR rats (for all, *P *< 0.05 or 0.001). Thus, inflammatory regulators (GDF-15, CAM-1, and VCAM-1) play critical roles in chronic inflammation pathogenesis in HFpEF rats.

### 3.4. Myocardial hypertrophy in HFpEF rat

**Figure 4:**
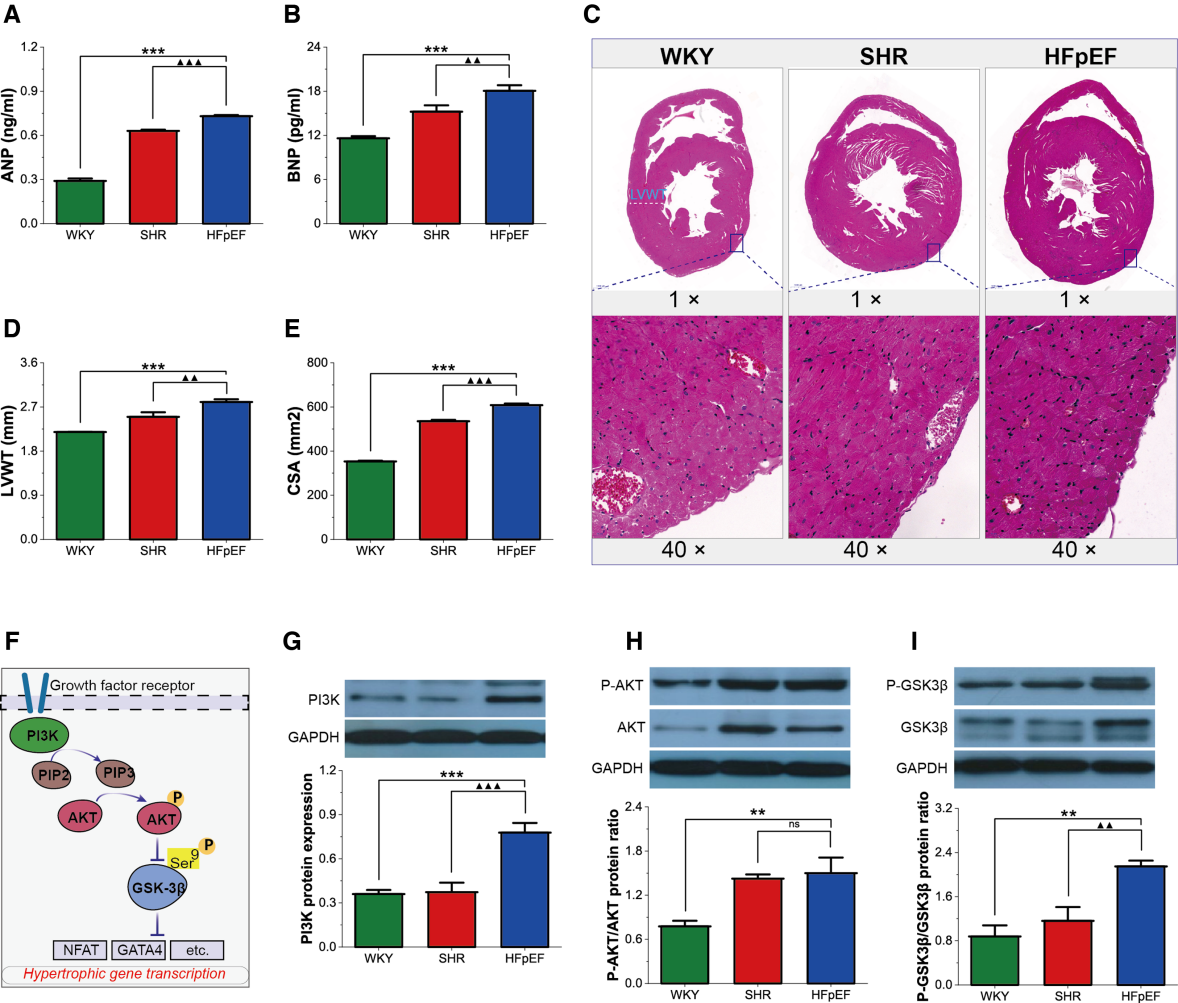
Myocardial hypertrophy in HFpEF rat. (**A,B**) Comparison of ANP and BNP between the three groups. (**C**) Representative images of HE-stained sections of LV myocardial tissue (top, 1×) and cross-sectional view of the LV cardiomyocytes (bottom, 40×): HFpEF rats exhibited significant LV hypertrophy compared with WKY and SHR rats, as evidenced by increased LV wall thickness and cardiomyocyte size compared to WKY and SHR rats. (**D,E**) Comparison of LVWT and CSA between the three groups. (**F**) AKT/GSK-3β signaling pathway in cardiac hypertrophy: On growth factor stimulation, PI3K phosphorylates PIP2 to PIP3, which recruits AKT to the plasma membrane, where AKT is activated in a phosphorylation-dependent manner. Activated AKT phosphorylates GSK-3β (Ser 9), leading to the inactivation of GSK3 activity and removing its inhibitory effect on cardiac hypertrophy. (**G–I**) Comparison of PI3K protein expression and the ratio of P-AKT/AKT and P-GSK-3β/GSK-3β protein between the three groups. Data are presented as mean ± standard deviations, analyzed with one-way ANOVA, *P < 0.05 vs. WKY;**P < 0.01vs WKY; ***P < 0.001vs WKY; ▴*P *< 0.05 vs. SHR, ▴▴*P *< 0.01 vs. SHR, ▴▴▴*P *< 0.001 vs SHR. ANP, atrial natriuretic peptide; BNP, B-type brain natriuretic peptide; LV, left ventricle; LVWT, LV wall thickness; CSA, cross-sectional area of LV cardiomyocytes; PI3K, phosphoinositide 3-kinase; PIP2, phosphatidylinositol-4,5-bisphosphate; PIP3, phosphatidylinositol-3,4,5- triphosphate; AKT, RAC-alpha serine/threonine-protein kinase; P-AKT, phosphorylated AKT; GSK3*β*, glycogen synthase kinase 3*β*; P-GSK3*β*, phosphorylated GSK3*β*. NFAT, nuclear factor of activated T-cells; GATA4, GATA-binding protein 4.

Pathological myocardial hypertrophy is one of the dominant changes in HFpEF. HFpEF rats demonstrated significant increases in serum hypertrophy markers (ANP and BNP), LVWT, and CSA compared with WKY and SHR rats (for all, *P* < 0.01 or 0.001, Figures 4A–E). Thus, HFpEF rats developed pathological myocardial hypertrophy, as shown by thickened LV walls and enlarged cardiomyocytes.

GSK-3β acts as a critical anti-hypertrophic regulator of cardiomyocytes. However, phosphorylation of the Ser9 site of GSK-3β, which is mainly regulated by the PI3K/AKT pathway, removes its inhibitory activity on hypertrophy and contributes to cardiomyocyte hypertrophy. In this study, PI3K protein expression, as well as the P-AKT/AKT and P-GSK-3β/GSK-3β protein ratios, were significantly higher in HFpEF rats than in WKY rats (for all, *P* < 0.01 or 0.001, Figures 4F–I), and PI3K protein expression and the P-GSK-3β/GSK-3β protein ratio were also higher than in SHR rats (for all, *P* < 0.01 or 0.001). The results suggested that the PI3K/AKT pathway might promote myocardial hypertrophy by regulating GSK-3β phosphorylation in HFpEF rats.

### 3.5. Myocardial fibrosis in HFpEF rat

**Figure 5:**
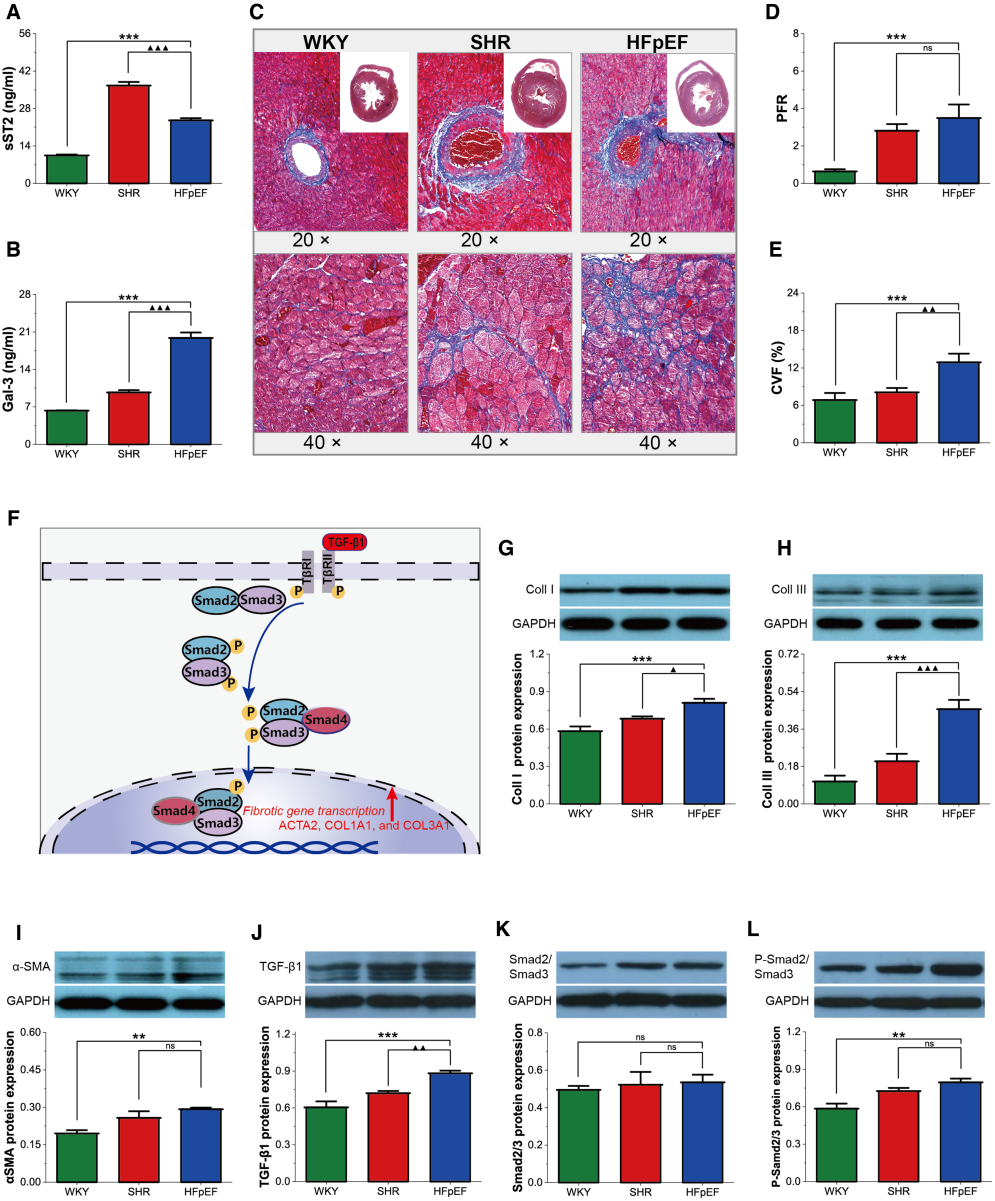
Myocardial fibrosis in HFpEF rat. (**A,B**) Comparison of serum sST2 and Gal3 between the three groups. (**C**) Representative images of Masson-stained sections of perivascular fibrosis (top, 20×) and LV interstitial (bottom, 40×): Masson trichrome staining showed significantly increased interstitial and perivascular fibrosis in HFpEF rats than in WKY and SHR rats. (**D,E**) Comparison of PFR and CVF between the three groups. (**F**) TGF-*β*1/Smads signaling pathway in cardiac fibrosis: TGF-*β*1 binds to its receptor and subsequently induces phosphorylation of the receptor-activated Smads (Smad2 and Smad3), which form trimeric complexes with the common-partner Smad (Smad4). The trimeric complexes translocate to the nucleus and then regulate the transcription of fibrotic genes, thereby modulating collagen synthesis and fibroblast activation, resulting in myocardial fibrosis. (**G**–**L**) Comparison of protein expression of Coll I, Coll III, *α*-SMA, TGF-*β*1, Smad2/3, and P-Smad2/Smad3 between the three groups. Data are presented as mean ± standard deviations, analyzed with one-way ANOVA, *P < 0.05 vs. WKY;**P < 0.01vs WKY; ***P < 0.001vs WKY; ▴*P *< 0.05 vs. SHR, ▴▴*P *< 0.01 vs. SHR, ▴▴▴*P *< 0.001 vs SHR. sST2, Soluble growth stimulated expression gene 2 protein, Gal-3, galectin-3; PFR, perivascular fibrosis; CVF, collagen volume fraction; *α*-SMA, a-Smooth muscle actin; Coll I, collagen type I; Coll III, collagen type III; TGF-*β*1, transforming growth factor-*β*1; P-Smad2/Smad3, phosphorylated Smad2/Smad3. ACTA2, actin alpha 2, smooth muscle; COL1A1, collagen type I alpha 1 chain; COL3A1, collagen type III alpha 1 chain.

Myocardial fibrosis is a well-recognized cause of LV diastolic dysfunction in HFpEF. In this study, serum biomarkers of fibrosis (sST2 and Gal-3), PFR, and CVF were statistically higher in HFpEF rats than in WKY rats (for all, *P* < 0.001, Figures 5A–E). Furthermore, although serum sST2 was lower in HFpEF rats than in SHR rats (*P* < 0.001), serum Gal3 and CVF were dramatically higher than in SHR rats (for all, *P* < 0.01 or 0.001). Therefore, HFpEF rats exhibited significant myocardial fibrosis, as shown by massive collagen deposition in the perivascular and myocardial interstitium.

The cardiac collagen mainly consists of Coll I and Coll III (11). Activated fibroblasts (marked by *α*-SMA) are responsible for collagen synthesis and deposition. In this study, Coll I, Coll III, and *α*-SMA protein expressions were considerably higher in HFpEF rats than in WKY rats (for all, *P* < 0.01 or 0.001, Figures 5G–I), and Coll I and Coll III protein expressions were markedly higher than in SHR rats (for all, *P* < 0.05 or 0.001). The findings indicated that myocardial fibrosis in HFpEF rats might be associated with fibroblast activation and excessive collagen deposition.

TGF-*β*1 activates the classical Smad signaling pathway, regulating downstream fibrosis-related genes, thereby modulating collagen synthesis and fibroblast activation, resulting in myocardial fibrosis (Figure 5F). The TGF-*β*1 and P-Smad2/Smad3 protein expressions were markedly higher in HFpEF rats than in WKY rats (for all, *P* < 0.01 or 0.001, Figures 5J,L), and TGF-*β*1 protein expressions were also higher than in SHR rats (for all, *P* < 0.01), suggesting that activating the TGF-*β*1/Smads pathway might regulate fibroblast activation and collagen synthesis, thereby promoting myocardial fibrosis in HFpEF rats.

## 4. Discussion

**Figure 6:**
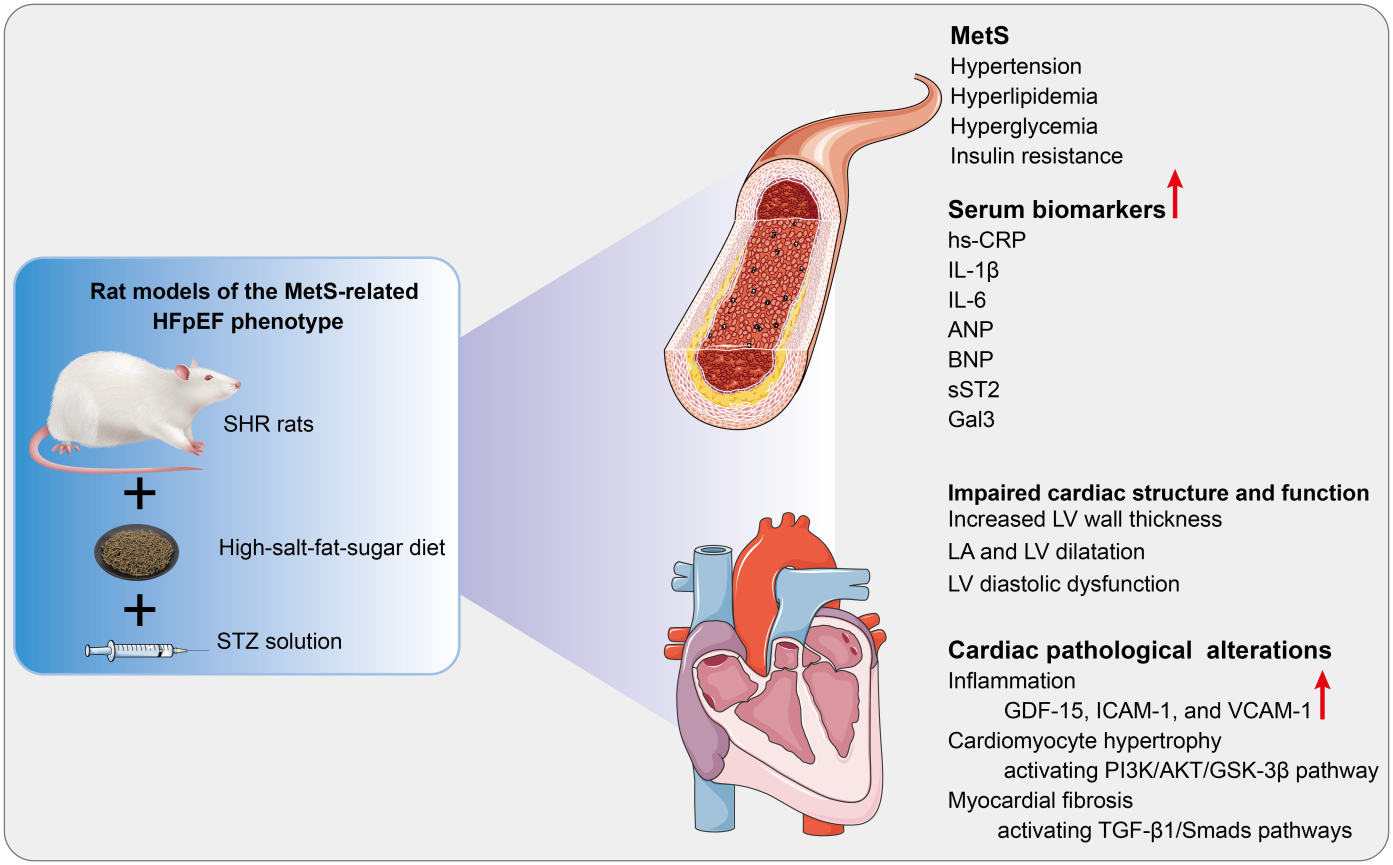
Cardiac pathological alterations and possible molecular mechanisms in HFpEF rat. A rat model of the MetS-associated HFpEF phenotype was established by feeding SHR rats a high-fat-salt-sugar diet and administering STZ solution intraperitoneally. The HFpEF rats demonstrated the primary features of MetS and HFpEF-related structural and functional cardiac abnormalities. Additionally, inflammation, myocardial hypertrophy, and fibrosis are observed in HFpEF rats, which may be associated with diverse cellular and molecular signaling cascades. MetS, metabolic syndrome; HFpEF, heart failure with preserved ejection fraction; SHR, spontaneously hypertensive rats; STZ: streptozotocin; hs-CRP, high-sensitivity C-protein; IL-1β, interleukin-1β; IL-6, interleukin-6; ANP, atrial natriuretic peptide; BNP, B-type brain natriuretic peptide; sST2, Soluble growth stimulated expression gene 2 protein, Gal-3, Galectin-3; LV, left ventricle; LA, left atrial; GDF-15, growth differentiation factor 15; ICAM-1, intercellular adhesion molecule-1; VCAM-1, vascular endothelial cell adhesion molecule-1. PI3K, phosphoinositide 3-kinase; AKT, serine/threonine protein kinase; GSK3*β*, glycogen synthase kinase 3*β*; TGF-*β*1, transforming growth factor-*β*1.

HFpEF is a complex disorder with various underlying pathophysiologies and significant phenotypic heterogeneity. Thus, targeting therapy based on phenotype and pathogenic mechanisms may be an effective strategy (2). An optimal animal model that accurately simulates the pathophysiological features of HFpEF can help elucidate the pathogenesis of HFpEF and explore therapeutic approaches. As shown in Figure 6, since MetS is a frequent comorbidity of HFpEF, a rat model of the MetS-associated HFpEF phenotype was established. The HFpEF rats exhibited the major characteristics of MetS and HFpEF-related structural and functional cardiac abnormalities. Moreover, inflammation, hypertrophy, and fibrosis were observed in the myocardium of HFpEF rats, which may be related to diverse cellular and molecular signaling cascades. These molecular mechanisms may involve the overexpression of inflammatory regulators (GDF-15, ICAM-1, and VCAM-1), activation of the AKT/GSK-3β pathway, and activation of the TGF-*β*1/Smads pathway, respectively.

### 4.1. Mets and abnormalities in cardiac structure and function in HFpEF rat

To date, there is no optimal preclinical model due to the complexity and heterogeneity of HFpEF. It is well-recognized that an “ideal” preclinical model of HFpEF is supposed to accurately reproduce the clinical phenotypes and pathophysiological features of HFpEF patients (12–14). MetS represents a primary comorbidity of HFpEF (15) and is strongly associated with the increased risk of all-cause hospitalization in HFpEF patients (16). Thus, developing animal models of the MetS-related HFpEF phenotype is critical. In the last decade, preclinical models of HFpEF with a single phenotype, like advanced age, hypertension, and glucolipid dysregulation, have been established. Marzak et al. and Danial et al. developed rat models of the aging or hypertension-related HFpEF phenotype by feeding SHR rats a regular diet for 28 and 36 weeks, respectively (12, 17). Dahl salt-sensitive rats developed HFpEF-related cardiac structure and function abnormalities after maintaining a high-salt diet for seven weeks, as reported by Zhang et al. (13). According to Wu et al., a mouse model of diabetes-associated HFpEF was induced by a combined high-fat diet with a low-dose STZ solution (18). Based on the methods mentioned above, a rat model of the MetS-related HFpEF phenotype was established by feeding SHR rats a high-fat-salt-sugar diet and injecting STZ solution intraperitoneally in this study.

The HFpEF rat simulated the clinical features of MetS, such as hypertension, glucolipid metabolism disorders, and insulin resistance. Additionally, HFpEF is a complex syndrome involving cardiac remodeling and LV diastolic dysfunction (19). LV diastolic dysfunction is marked by prolonged isovolumic relaxation, impaired relaxation, reduced compliance, and elevated LV filling pressures, which are usually assessed by the echocardiographic parameters of IVRT, e′, a′, and E/e′, respectively (20, 21). The HFpEF rats in this study developed cardiac remodeling and LV diastolic dysfunction. Compared with ZSF-1 rats, a currently available rat model of the MetS-related HFpEF phenotype, the HFpEF rats established in this study mimic both the genetic predisposition to hypertension and the multiple complex comorbidities of aging and long-term glucolipid metabolism disorders, thereby better replicating human HFpEF pathogenesis.

### 4.2. Systemic and myocardial inflammation in HFpEF rat

A systemic pro-inflammatory state might represent a bridge between various comorbidities and impaired cardiac structure or function in HFpEF (22). MetS-induced inflammation may trigger various pathophysiological changes, including myocardial hypertrophy or fibrosis (10, 23). According to Sanders-van Wijk et al., systemic inflammation might regulate the correlation between comorbidity and echocardiographic indices related to hemodynamic deterioration and LV diastolic dysfunction (24). Additionally, serum inflammation biomarkers, such as hs-CRP, IL-6, and GDF-15, have been shown to strongly associate with severity and adverse outcome in HFpEF patients (22, 25, 26). Furthermore, marked inflammatory cell infiltration was identified in endomyocardial biopsy specimens from HFpEF patients, indicating that HFpEF may represent a chronic inflammatory syndrome (27). Our study confirmed that HFpEF rats exhibited systemic and myocardial inflammation.

Inflammation involves diverse inflammatory mediators, such as cytokines, cell adhesion molecules, and chemokines. GDF-15 is an inflammatory cytokine secreted in response to inflammation and exerts an essential role in the pathophysiology of HFpEF (28). Cell adhesion molecules, especially ICAM-1 and VCAM-1, mediate the adhesion of circulating leukocytes to the endothelium and subsequent migration to the arterial wall, which is an early stage of inflammation (29). A significant increase in ICAM-1 and VCAM-1 protein expression has been reported in the myocardium of HFpEF patients and ZSF-1 rats (30, 31). In this study, overexpression of GDF-15, ICAM-1, and VCAM-1 proteins was observed in HFpEF rats, suggesting that these three inflammatory mediators may be involved in chronic inflammation of the myocardium in HFpEF rats. Pharmacological blockage of ICAM-1 and VCAM-1 was confirmed to alleviate hypertensive cardiac remodeling by regulating the adhesion and infiltration of monocytes and macrophages (32–35). Accordingly, blocking ICAM-1 or VCAM-1 might be a promising therapeutic strategy for HFpEF and deserves further confirmation in relevant studies.

### 4.3. Pathological cardiac hypertrophy in HFpEF rat

Pathological myocardial hypertrophy, mainly defined by LV hypertrophy (LVH) and cardiomyocyte enlargement, represents an adaptive reaction of the heart to elevated hemodynamic pressure (36). LVH, presenting in approximately 30%–60% of HFpEF patients, is a significant contributor to LV diastolic dysfunction and elevated diastolic filling pressures [5]. Histological analysis of the hearts of HFpEF patients revealed significant pathological cardiac hypertrophy and enlarged cardiomyocytes (37). Furthermore, LVH was found to be independently related to all-cause death and HF hospitalization in individuals with HFpEF, regardless of clinical predictors and measures of diastolic function (38). In this study, HFpEF rats developed marked LV wall thickening and cardiomyocyte enlargement.

Increasing evidence indicates that GSK-3β exerts an anti-hypertrophic regulatory role in cardiomyocytes. GSK-3β shows high activity in the basal condition (i.e., unstimulated cells) and inhibits various pathways that promote cardiac hypertrophy (for example, GSK-3β phosphorylates the nuclear factor of activated T-cells and induces its nuclear export) (39). However, growth factors, like insulin, which stimulate the PI3K/AKT pathway, may deactivate GSK-3 by phosphorylating the N-terminal serine residue at Ser-9 (40–42). Therefore, the activated PI3K/AKT pathway may suppress the activity of GSK-3β, thereby removing an inhibitory constraint on pro-hypertrophic pathways. In this study, cardiac hypertrophy in HFpEF rats may be associated with PI3K/AKT signaling pathway activation, further inhibiting GSK-3β activation. Promisingly, inhibiting the AKT/GSK-3β pathway may attenuate myocardial hypertrophy induced by pressure overload (43–45). Thus, targeted inhibition of GSK-3β may provide clinical benefits in treating pathologic hypertrophy in HFpEF.

### 4.4. Myocardial fibrosis in HFpEF rat

Myocardial fibrosis, characterized by perivascular and interstitial fibrosis, is an essential pathological feature of HFpEF (46, 47). Hahn et al. performed endomyocardial biopsies on 108 HFpEF patients, confirming that about 93% of individuals showed evidence of fibrosis (48). Notably, myocardial fibrosis is responsible for passive muscle stiffening, reduced chamber compliance, and LV diastolic dysfunction in HFpEF patients. As reported, extracellular volume (ECV) and indexed ECV (iECV), the metrics of diffuse myocardial fibrosis, were linked to invasively measured LV stiffness and LV diastolic dysfunction in HFpEF patients, respectively (49, 50). Furthermore, indicators of myocardial fibrosis, such as serum sST2 and Gal3, ECV, and iECV, were shown to be independent predictors of unfavorable endpoints in HFpEF patients (51–54). In this study, HFpEF rats exhibited significant perivascular and myocardial interstitial fibrosis.

Cardiac collagen, primarily comprising 85% Coll I and 11% Coll III, is the major structural protein of the cardiac extracellular matrix (ECM) (11). Myofibroblasts, a subpopulation of activated fibroblasts characterized by the expression of *α*-SMA, are responsible for collagen production and deposition. We confirmed a significant increase in myofibroblasts and ECM production in HFpEF rats. The TGF-*β*1/Smads pathway serves as an essential modulator of collagen synthesis and fibroblast activation. TGF-*β*1 binds to its receptor and induces phosphorylation of the receptor-activated Smads (Smad2 and Smad3), which combine with the common-partner Smad (Smad4) to create trimeric complexes. The trimeric complexes migrate to the nucleus and then modulate fibrosis-associated gene transcription, including ACTA2, COL1A1, and COL3A1, which encode *α*-SMA, Coll I, and Coll III, respectively (55). In this study, TGF-*β*1/Smads pathway activation may regulate fibroblast activation and ECM synthesis, thereby promoting myocardial fibrosis in HFpEF rats. Dapagliflozin ameliorates angiotensin II-induced myocardial fibrosis by suppressing TGF-*β*1/Smads pathway (56). Consequently, although no specific anti-fibrotic drugs are routinely used in HFpEF treatment, targeting the TGF-*β*1/Smads pathway may be a possible therapeutic strategy to alleviate myocardial fibrosis in HFpEF and deserves further investigation.

In conclusion, The HFpEF rat model simulates critical characteristics of human HFpEF from pathology to clinic and thus is a reliable preclinical model of HFpEF. Nevertheless, there are corresponding shortcomings that deserve to be considered. First, the rat model may not fully mimic the human syndrome, as HFpEF is a heterogeneous disorder associated with multiple risk factors and cardiac or extra-cardiac pathological alterations. Second, LVEF in SHR rats may decrease with age, especially older than 15 months. Therefore, studies that observed diastolic dysfunction mainly used SHR rats younger than 15 months and dynamically observed cardiac structure and function changes. In future experiments, we will dynamically examine the cardiac structure and function of the rats. Moreover, serum hs-CRP, IL-6, and sST2 in SHR rats were markedly higher than in HFpEF rats, which may be related to limited sample size, specimen contamination, or HFpEF rats receiving multiple risk factor interventions. We will further explore this in future experiments. Finally, further *in vitro* experiments are still needed to validate these potential molecular mechanisms.

## 5. Conclusions

A rat model of the MetS-associated HFpEF phenotype was established by feeding SHR rats a high-fat-salt-sugar diet and administering STZ solution intraperitoneally. The HFpEF rats demonstrated the primary features of MetS and HFpEF-related structural and functional cardiac abnormalities. Additionally, inflammation, myocardial hypertrophy, and fibrosis are observed in HFpEF rats. These pathological changes may involve overexpression of inflammatory regulators (GDF-15, ICAM-1, and VCAM-1), activation of the AKT/GSK-3β pathway, and activation of the TGF-*β*1/Smads pathway, respectively. Consequently, the HFpEF rat replicates critical features of human HFpEF, including pathology and clinical presentation, and thus may be a reliable preclinical model that may help elucidate HFpEF pathogenesis and explore potential therapeutic interventions.

## Data Availability

The datasets presented in this study can be found in online repositories. The names of the repository/repositories and accession number(s) can be found below: https://figshare.com/s/bb4611a46fd19e614d7f.
