# Supplementary material for: A rat model of metabolic syndrome-related heart failure with preserved ejection fraction phenotype: pathological alterations and possible molecular mechanisms
Source: Front Cardiovasc Med. 2023 Jul 4;10:1208370. doi: 10.3389/fcvm.2023.1208370 (PMC10352810; doi:10.3389/fcvm.2023.1208370)
Supplement: Supplementary file 1 [file Table1.docx]

Supplementary Material

| **Antibody names** | **Antibody source** | **Catalogue number** | **Producers** |
| --- | --- | --- | --- |
| anti-GDF-15 | Rabbit | ab39999 | Abcam, United Kingdom |
| anti-ICAM-1 | Rabbit | GTX100450 | GeneTex, America |
| anti-VCAM-1 | Rabbit | ab134047 | Abcam, United Kingdom |
| anti-PI3K | Rabbit | GTX111068 | GeneTex, America |
| anti-AKT | Rabbit | 9272 | GST,America |
| anti-P-AKT | Rabbit | 4056 | GST,America |
| anti-GSK3β | Rabbit | 12456 | GST,America |
| anti-P-GSK3β | Rabbit | 5558 | GST,America |
| anti-Coll I | Rabbit | bs-0578 | Bioss, America |
| anti-Coll III | Rabbit | bsm-33129 | Bioss, America |
| anti-α-SMA | Rabbit | ab53219 | Abcam, United Kingdom |
| anti-TGF-β1 | Rabbit | ab92486 | Abcam, United Kingdom |
| anti-Smad2/Smad3 | Rabbit | ab202445 | Abcam, United Kingdom |
| anti-P-Smad2/Smad3 | Rabbit | ab272332 | Abcam, United Kingdom |
| anti-GAPDH | Mouse | YM3029 | Immunoway, America |

Supplementary Table 1. The characteristics of the antibodies included in the study

**Abbreviations:** GDF-15, growth differentiation factor 15; ICAM-1, intercellular adhesion molecule-1; VCAM-1, vascular endothelial cell adhesion molecule-1; PI3K, phosphoinositide 3-kinase; AKT, RAC-alpha serine/threonine-protein kinase; P-AKT, phosphorylated AKT; GSK3β, glycogen synthase kinase 3β; P-GSK3β, phosphorylated GSK3β; Coll I, type I collagen; Coll III, type III collagen; α-SMA, Α-Smooth muscle actin; TGF-β1, transforming growth factor-β1; P-Smad2/Smad3, phosphorylated Smad2/Smad3; GAPDH, glyceraldehyde-3-phosphate dehydrogenase.
